# Comparative evaluation of the effect of Er:YAG laser and low level laser irradiation combined with CPP-ACPF cream on treatment of enamel caries

**DOI:** 10.4317/jced.51309

**Published:** 2014-04-01

**Authors:** Farzin Heravi, Farzaneh Ahrari, Mahdieh Mahdavi, Soroush Basafa

**Affiliations:** 1 DDS MS, Associate Professor of Orthodontics. Dental Research Center, School of Dentistry, Mashhad University of Medical Sciences, Mashhad, Iran; 2 DDS MS, Assistant Professor of Orthodontics. Dental Research Center, School of Dentistry, Mashhad University of Medical Sciences, Mashhad, Iran; 3 DDS, Private practice. Mashhad, Iran

## Abstract

Objectives: This study investigated the effectiveness of low power red and infrared lasers and that of Er:YAG laser, in association with CPP-ACPF cream, on remineralization of white spot lesions. Study Design: Fifty intact premolars were immersed in a demineralization solution for 10 weeks to induce caries like lesions and then were divided into five groups. In group 1, the teeth were covered with a CPP-ACPF cream for 3 minutes and then irradiated with a low power red laser (660 nm, 200 mW) for 1 minute through the cream. In group 2, the treatment was the same as that in group 1, but an infrared laser (810 nm, 200 mW) was employed. The specimens in group 3 were irradiated with an Er:YAG laser (100 mJ, 10 Hz) combined with CPP-ACPF. In group 4, the CPP-ACPF cream was applied for 4 minutes and group 5 was submitted to neither laser nor CPP-ACPF. The micro Vickers hardness was compared at 20, 60 and 100 µ from the enamel surface among the groups. Results: The highest microhardness was observed in the low power red and Er:YAG laser groups and the lowest one belonged to the CPP-ACPF alone and control groups. However, no significant difference was found in microhardness of the experimental groups at any of the evaluation depths (p>0.05). Conclusions: With the laser parameters used in this study, neither the combined application of Er:YAG laser with CPP-ACPF nor the combination of low power lasers with CPP-ACPF provided a significant increase in remineralization of enamel caries.

** Key words:**Low level laser, Er:YAG, laser, enamel caries, CPP-ACP, microhardness, white spot lesion.

## Introduction

Dental caries is still the most common oral disease throughout the world (1). Enamel decalcification or the for-mation of white spot lesions is the first sign of dental caries usually appeared as chalky white areas on the tooth surface. This condition, which jeopardizes tooth health and esthetics, is frequently observed in patients with poor oral hygiene and high levels of caries activity especially those undergoing fixed orthodontic treatment, because appliances accumulate food debris and dental plaque. If suitable treatment is presented to these lesions, enamel caries is capable to arrest, reharden and reverse to the healthy enamel condition via a remineralization process involving the diffusion of minerals into the defective tooth structure. The remineralization treatment is usually performed with fluoride or other prophylactic agents, which can be combined with lasers to promote their effects (2,3).

Recently, casein phosphopeptide amorphous calcium phosphate (CPP-ACP) products have been introduced for prevention of dental caries and induction of remineralization in tooth tissues (4,5). Recent studies recognized that incorporation of fluoride into the CPP-ACP structure (CPP-ACPF) produced greater remineralization than the CPP-ACP alone (6-8). There are few studies regarding the effectiveness of this agent on remineralization of white spot lesions and no study to date investigated the anti-caries benefits of CPP-ACPF cream combined with low- or high- power lasers.

It has been demonstrated that the application of high power lasers such as CO2 and erbium lasers (Er:YAG and Er,Cr:YSGG) are effective in caries prevention (9-13). As these lasers are greatly absorbed by water and hy-droxyapatite of the tooth tissues, they are capable to modify the crystalline structure, acid solubility and permeability of the tooth surface and thereby increasing its resistance against demineralization. The high power lasers, however, are costly and not readily available in every practice. Low level red and infrared lasers are relatively inexpensive, small and transmittable and have multiple applications in several brands of dentistry, so their application for prevention or arrestment of dental caries seems interesting. The outcomes of previous studies on caries preventive effects of low power lasers, however, are controversial (1,14-15) and the efficacy of these lasers for promoting enamel remineralization has not been sufficiently investigated.

The aim of this study was to investigate the effectiveness of low power red and infrared lasers and that of the Er:YAG laser, in association with CPP-ACPF cream, on remineralization of white spot lesions of enamel.

## Material and Methods

Fifty freshly extracted maxillary premolar teeth were collected and stored in saline solution at room temperature until the time of the experiment. Teeth with caries, cracks and defects in enamel structure were discarded. The specimens were immersed in a demineralizing solution containing 2.2 mM CaCl2, 2.2 mM NaH2PO4, and 50 mM acetic acid (PH 4.8) (16) for 10 weeks to induce white spot lesions. The teeth were then coated with two conse-cutive layers of nail varnish except for a 4×4 mm window which was exposed at the center of the buccal surface to serve as the treatment area. After that, the specimens were randomly divided into five experimental groups according to the procedure used for the treatment of demineralized enamel.

Group 1 (low power red laser; LPRL): In this group, the teeth were covered with CPP-ACPF cream for a total period of 4 minutes. After 3 minutes of cream application, the enamel was irradiated with a low power red laser (Thor DD2 Control Unit, Thor, London, UK) for 1 minute, and the cream was allowed to remain on the surface during irradiation. The laser apparatus emitted a wavelength of 660 nm and operated at the maximum power of 200 mW, continuous wave mode and at the approximate distance of 5 mm from the tooth surface. The total energy delivered to the enamel was 6 J.

Group 2 (low power infrared laser; LPIL): The demineralized enamel was covered with CPP-ACPF cream for four minutes and was irradiated with a low power infrared laser at the last one minute of cream application, similar to that described for group 1. The device was a gallium aluminum arsenide (Ga-Al-As) diode laser (Thor DD2 Control Unit, Thor, London, UK), emitting a wavelength of 810 nm and operating at the maximum output power of 200 mW in continuous wave mode. Each treatment window received 6 J of energy.

Group 3 (Er:YAG laser): The treatment windows in this group were first covered with a thin layer of CPP-ACPF cream for 30 seconds, and then the Er:YAG laser (wavelength 2940 nm; Smart 2940 D, Deka Laser, Firenze, Italy) was irradiated through the cream over a period of 20 s. The handpiece was held manually at an approximate distance of 5 mm (focused mode) and perpendicular to the tooth surface, and the area was irradiated uniformly using scanning movements. The laser beam was emitted with energy of 100 mJ, frequency of 10 Hz, and with the use of minimum air/water spray. The beam diameter at the focal area of the handpiece was 1 mm, providing the energy density (fluence) of 12.7 J/cm2 per pulse. After laser irradiation, the thickness of the CPP-ACPF cream was increased as necessary and it was allowed to remain for a total period of 4 minutes on the enamel surface.

Group 4 (CPP-ACPF alone): The demineralized enamel in this group was only treated with application of a sufficient thickness of CPP-ACPF cream for 4 minutes.

Group 5 (Control): The teeth in the control group were not subjected to either CPP-ACPF cream or laser irradia-tion.

After the treatment procedures in the experimental groups, the CPP-ACPF cream was thoroughly rinsed from the enamel surfaces, and the teeth were then kept in distilled water until microhardness assessment.

For microhardness analysis, the crowns were separated from the roots and then were embedded in epoxy resin. The specimens were sectioned occlusogingivally through the center of the treatment windows with a diamond disk at low speed, and the enamel surfaces were polished with sandpaper discs. After rinsing with distilled water, microhardness was assessed by a micro Vickers hardness apparatus (Matsuzawa, model MHT2, Japan) using a vertical load of 100 g for 5 seconds. The indenter of the apparatus was placed at 20 µ, 60 µ and 100 µ from the enamel surface to determine Vickers hardness number (VHN) at different depths.

- Statistical analysis

The normal distribution of the data was confirmed by the Kolmogorov-Smirnov test and the homogeneity of variances by the Levene’s test. A one-way ANOVA was run to determine the difference in microhardness of the experimental groups at each enamel depth. The statistical analysis was done using Statistical Package for the Social Sciences (version 16.0, SPSS Inc., Chicago, IL, USA) and the significance level was determined at *p*<0.05.

## Results

 Table 1,  Table 2 and  Table 3 present the descriptive statistics and the results of statistical analyses for comparison of microhardness values among groups at 20 µ, 60 µ and 100 µ from the surface, respectively. Generally, the highest microhardness values were observed in the LPRL and Er:YAG laser groups and the lowest ones belonged to the CPP-ACPF alone and control groups. The mean microhardness of the specimens treated with CPP-ACPF alone was very close to that obtained in the control group. ANOVA displayed no significant differences in microhardness of the experimental groups at any of the evaluation depths (*p*>0.05;  Table 1- Table 3).

**Table 1:**
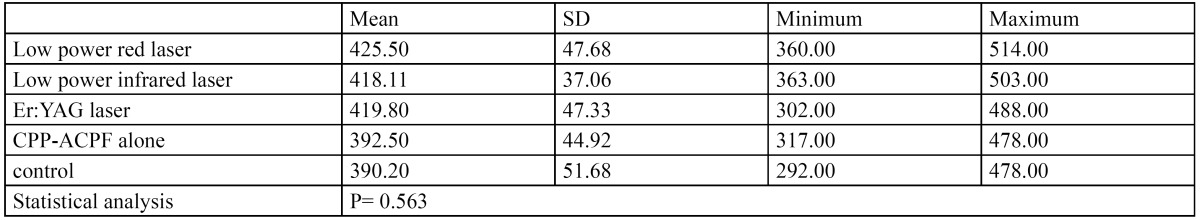
The descriptive statistics and the results of statistical analysis for comparison of microhardness values at 20 µ depth among the study groups.

**Table 2:**
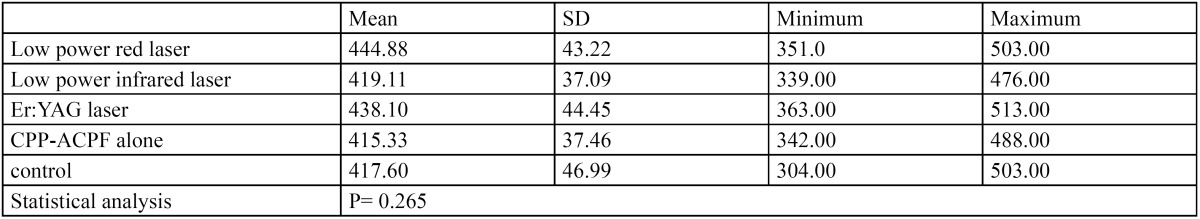
The descriptive statistics and the results of statistical analysis for comparison of microhardness values at 60 µ depth among the study groups.

**Table 3:**
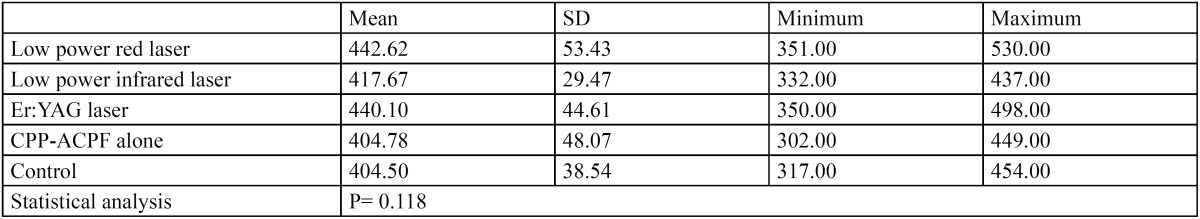
The descriptive statistics and the results of statistical analysis for comparison of microhardness values at 100 µ depth among the study groups.

## Discussion

Although fluoride is the most effective agent in prevention and regression of dental caries, its repeated applica-tion may be associated with the risk of dental fluorosis (17). Caries prevention and inhibition can also be achieved with CPP-ACP cream, which is a milk-derived product. The CPP part of this complex is used to localize amorphous calcium phosphate (ACP) in dental plaque and provide a state of supersaturation with respect to enamel (5,18). The present study stands as the first to investigate the effectiveness of fluoride-incorporated CPP-ACP complex (CPP-ACPF) in combination with low power (red and infrared) and Er:YAG lasers on remineralization of enamel white spot lesions.

In this study, the microhardness of the specimens treated with the CPP-ACPF cream was almost the same as those untreated. This means that one time application of this agent had no effect on remineralization of white spot lesions. It has been demonstrated that CPP-ACP products bind to dental plaque and provide a source of readily available calcium that restricts mineral loss and assists remineralization (5,18). Some in vivo studies (19-20) indicated the beneficial effects of CPP-ACP cream on remineralization of white spot lesions compared to the control group, but in these studies, the cream was applied daily for 1 or 3 months. The extended exposure times or frequent applications of CPP-ACP products may produce beneficial effects on in vitro remineralization of enamel caries, but further studies are warranted to verify these issues.

Different theories have been explained for laser-induced changes in tooth structure. Regarding the high power lasers, it is believed that the photothermal effects of lasers are responsible for the modification in dental hard tissue structure including reduction of carbonate content and alterations in organic matters which lead to increase in the acid resistance (21). The mechanism of low level lasers in caries prevention may be related to their photochemical effects, because laser wavelengths at the range of 450-800 nm have low absorption in enamel (1). Some authors (1,14) proposed that application of a photoabsorbing cream on the enamel surface, which should be proportional to the wavelength of the low power laser, can efficiently intensify energy absorption and transform it to heat, thus providing photothermal in addition to the photochemical changes in the enamel surface and enhancing its acid resistance.

In this study, the application of Er:YAG laser with fluence of 12.7J/cm2 caused 7% increase in microhardness at 20 µ depth and about 4% and 8% increase at 60 µ and 100 µ depths, respectively, compared to the control group. Although this laser provided some beneficial effects in caries treatment, the overall benefit was small and statistically insignificant. This finding corroborates the results of some previous authors (22-24) who observed no significant increase in caries resistance of the specimens irradiated with the Er:YAG or Er,Cr:YSGG lasers compared to the control group. The outcomes of this study, however, are in contrast to the studies that indicated caries inhibitive effects of erbium lasers (11-13). de Freitas *et al.* (12) reported that the specimens submitted to the Er,Cr:YSGG laser at 8.5 J/cm2 showed 64% caries inhibition which was comparable to the cariostatic effect of the samples treated with a NaF dentifrice. Bevilacqua *et al.* (11) employed a wide range of energy densities of Er:YAG laser followed by fluoride treatment and reported that the higher fluoride incorporation in enamel occurred in the samples irradiated with the lowest fluencies (1.8 J/cm2 and 0.9 J/cm2), whereas a reduction in calcium solubility was observed in the specimens irradiated with the highest laser fluence (31.84 J/cm2).

The discrepancy observed between the outcomes of this study and those of previous authors may be related to the different laser parameters including pulse energy, pulse duration, repetition rate, energy density, and period of irradiation, or to the application of water and air cooling during treatment. We used the lowest laser parameters possible with the apparatus used in this study in order to reduce the risk of ablation and enamel cracks. A small amount of air and water cooling was also employed to minimize any detrimental effect of laser irradiation including carbonization on the porous structure of demineralized enamel (25). Hossain *et al.* (26), however, indicated that Er:YAG laser without water mist was more effective in caries prevention than when it was used with water coolant. This has also been emphasized by other authors (13) who found that water, even in minimal amounts, was not favorable for the preventive effect of laser irradiation.

The specimens irradiated with low power red laser showed 8% increase in microhardness at 20 µ depth and about 6% and 8.5% increase at 60 µ and 100 µ depths, respectively, compared to the control group. The remineralization effect of infrared laser was lower, as it caused 6% increase at 20 µ depth and about 3-4% increase at the underlying layers. Although the application of low power red laser through the CPP-ACPF cream produced the highest microhardness among the study groups, its overall benefit in remineralization of white spot lesions failed to achieve statistical significance. These findings are in agreement with those of Muller *et al.* (15) who investigated the effect of low power red laser (LPRL) irradiation on prevention of dental caries in rat molars and reported that neither the microhardness assay nor the EDS analysis revealed any advantageous effect of laser treatment on enamel. In contrast, de Sant’anna *et al.* (1) observed reduction and modification in the organic matrix of enamel induced by low level laser irradiation. In another study, these authors (14) reported that low level laser irradiation combined with a photoabsorbing cream was able to preserve the calcium and phosphorus content of enamel during cariogenic challenges and increase the inorganic/organic ratio of dental enamel with a significant difference to the control group. Vlacic *et al.* (27) observed that the combined application of seven different laser wavelengths (both visible and near-infrared) with fluoride protected the enamel surface from a strong erosion-like challenge.

It should be noted that most of the previous studies on cariostatic effects of erbium or low power lasers used laser beam for caries prevention and not for the treatment of demineralized enamel (1,3,11,14). These studies also applied laser in combination with high concentration of fluoride-containing agents and not with the CPP-ACPF cream (1,3,11,14). Since the CPP-ACPF had no effect on enamel remineralization in this study, it was expectable that its combined application with laser would provide no synergistic effect. Furthermore, the presence of CPP-ACPF on the tooth, which was applied to reduce any adverse structural effects of laser on the enamel, may prevent to some extent from temperature increase at the tooth surface and thus limitting the rehardening effect of the laser, as observed in a previous study with a fractional CO2 laser (10).

The laser effect on enamel morphology is an important subject that should be taken into account during treatment. It is possible that the increase in caries resistance be taken at the expense of inducing cracks and rougher enamel surface, which can make the tooth more susceptible to adhesion of bacterial plaque and acid attack (28).The detrimental effects may be greater on demineralized versus healthy enamel and may be observed when using high-power lasers such as Er:YAG laser, while low power laser irradiation should be considered safe regarding the effects on enamel surface morphology. The increase in temperature of the pulp and underlying layers is another subject of interest, although previous studies reported minimal temperature increases in the pulp either with the application of erbium lasers (29) or with low power laser irradiation (27).

The characteristics of the laser beam including wavelength, power, energy density, pulsed or continuous emis-sion, and other factors such as duration of irradiation and mode of application have great effects on the treatment results, so further studies are suggested to find the optimal laser parameters for the treatment of demineralized enamel without detrimental effects on surface morphology.

## Conclusion

The application of CPP-ACPF cream for 4 minutes on the tooth surface had no effect on remineralization of carious enamel compared to the control group.

Despite the highest microhardness observed in the Er:YAG and LPRL groups, with the parameters used in this study, neither the combined application of Er:YAG laser with CPP-ACPF cream nor the combination of low power lasers (red or infrared) with CPP-ACPF cream provided a significant increase in remineralization of enamel white spot lesions.
